# Editorial: Coronavirus Disease (COVID-19): Diet, Inflammation and Nutritional Status

**DOI:** 10.3389/fnut.2021.760720

**Published:** 2021-09-14

**Authors:** Ioannis Zabetakis, Christophe Matthys, Alexandros Tsoupras

**Affiliations:** ^1^Department of Biological Sciences, University of Limerick, Limerick, Ireland; ^2^Bernal Institute, University of Limerick, Limerick, Ireland; ^3^Health Research Institute, University of Limerick, Limerick, Ireland; ^4^Clinical and Experimental Endocrinology, Department of Chronic Diseases and Metabolism, KU Leuven, Leuven, Belgium; ^5^Department of Endocrinology, University Hospitals Leuven, Leuven, Belgium

**Keywords:** inflammation, chronic disease, COVID-19, obesity, nutrition, Mediterranean diet (MD)

Since late 2019, the world has come to terms with the fact that it is facing a major public health crisis against the novel coronavirus disease termed COVID-19 caused by the severe acute respiratory syndrome coronavirus 2 (SARS-CoV-2). As of August 12th 2021, there are over 205 million confirmed cases of COVID-19 and 4,300,000 deaths globally while more than 180 million patients have recovered. The first known case of COVID-19 originated from the city of Wuhan in Hubei Province, China. From there, it has spread to every inhabited continent worldwide.

COVID-19 is a highly infectious disease with severe or even lethal complications and a significant impact on the way in which we live our lives. Whilst the vast majority of people infected with COVID-19 experience mild symptoms and can recover without the need for hospitalization, it has become increasingly clear that those with pre-existing non-communicable diseases such as chronic lung diseases, metabolic syndrome, obesity and diabetes mellitus, cardiovascular disease, and renal disorders, as well as people under immunosuppression (i.e., the elderly and people with chronic rheumatoid conditions like rheumatoid arthritis, Lupus, etc.), are at increased risk of severe illness and mortality. The inflammatory and thrombo-inflammatory manifestations and cascades following SARS-CoV-2 infection are directly linked to the increased severity of COVID-19 complications, especially in patients with such underlying conditions and the elderly.

Thus, the potential for specific foods and nutrients that can beneficially affect COVID-19 severity and outcomes is gathering increasing interest from the scientific community, as well as the general population and media. Given that a common complication in patients with severe COVID-19, and individuals with non-communicable diseases, is excessive inflammation, foods with anti-inflammatory properties may possess a protective role. Likewise, the role of nutritional status and other nutrients, such as selenium, vitamin D, and vitamin C has gathered attention, with vitamin D and selenium deficiency recently linked to COVID-19 severity. Nevertheless, the potential for supplementation of specific foods and nutrients to act as a protective measure against COVID-19 remains a topic of debate.

The goal of this Research Topic was to gather Original Research articles and Reviews which have improved our understanding of diet, specific foods and nutritional status in relation to COVID-19 severity and outcomes. Twenty-eight articles have been published under this Research Topic covering a wide variety of Covid-19, the role of nutrition and how specific nutrients may be able to inhibit the spread of the disease and reduce its detrimental health effects. The articles addressed several aspects of Covid-19 from a metabolic, exercise, and nutrition point and they inform the readers on the latest developments of research related to Covid-19. The articles include perspectives, review articles and research articles and they also address the covid-19 related inflammation and cytokine storm.

The role of specific nutrients is widely discussed in this Research Topic from this inflammation point of view for this disease. It was also described that apart from inflammation, thrombosis, and thrombo-inflammatory complications are also linked to COVID-19, and the role of exercise and diet is discussed further. The role of foods, minerals, and vitamins is presented as means to reduce the cytokine storm and covid-19 related inflammation. The impact of lifestyle is also presented. The role of vitamin C as a potential aid in the treatment of Covid-19 is discussed in Cerullo et al. Also, the role of vitamins C and D and zinc are discussed in relation to physical tissue barrier integrity in Name et al.

The role of food supplements in relation to immune-boosting, antioxidant, and anti-inflammatory activities is presented in relation to Covid-19 pathogenesis. This article Mrityunjaya et al., suggests that grouping phytonutrients in the form of a food supplement may help to boost the immune system, prevent virus spread, preclude the disease progression to severe stage, and further suppress the hyper inflammation providing both prophylactic and therapeutic support against COVID-19.

The stormy link between obesity and Covid-19 is presented in López-Reyes et al. The authors have concluded that the SARS-CoV-2 infection could potentiate or accelerate the pre-existing systemic inflammatory state of individuals with obesity, *via* the NLRP3 inflammasome activation and the release of pro-inflammatory cytokines from cells trough Gasdermin-pores commonly found in cell death by pyroptosis.

In Bennett et al., it was found that the effect of COVID-19 lockdown both negatively and positively impacted dietary practices throughout Europe and globally, and negative diet habits were associated with other poor lifestyle outcomes including weight gain, mental health issues, and limited physical activity. Both in the short term and if sustained in the long term, these changes may have significant impacts on the health of the population.

In Vassilopoulou et al., an article linking breastfeeding and covid-19, it was highlighted that since SARS-CoV-2 is highly transmissible, breastfeeding should be encouraged, but observing all appropriate safety measures, for the mother and close contacts is needed. With the vaccination schedule in progress, the pregnant mothers-to-be should probably be prioritized.

In Zaragoza-Martí et al., an article linking Mediterranean lifestyle and lockdown, it was found that adherence to the Mediterranean Diet may play an important role in the establishment of appropriate dietary guidelines in confinement situations such as the COVID-19.

People with underlying conditions are more susceptible to COVID-19; as a matter of fact around 95% of COVID-19 related deaths are people with severe chronic diseases and these data manifest that COVID-19 is a deadly virus in people with chronic inflammation. In other words, a pre-existing chronic inflammatory status detrimentally affects our immune system and homeostatic mechanisms, and thus make us more susceptible to the cytokine storm and its associated inflammatory manifestations that COVID-19 causes in our bodies. The potential role of nutrition in relation to thrombosis and COVID-19 19 is discussed in Tsoupras et al.

In conclusion, this Research Topic, with a variety of articles has provided, a wealth of insights on the link between nutrition, inflammation, and covid-19, with promising outcomes and future perspectives. As Editors, we would like to thank all the contributing authors and the people in Frontiers in Nutrition for their excellent editing support.

## Author Contributions

All authors listed have made a substantial, direct and intellectual contribution to the work, and approved it for publication.

## Conflict of Interest

The authors declare that the research was conducted in the absence of any commercial or financial relationships that could be construed as a potential conflict of interest.

## Publisher's Note

All claims expressed in this article are solely those of the authors and do not necessarily represent those of their affiliated organizations, or those of the publisher, the editors and the reviewers. Any product that may be evaluated in this article, or claim that may be made by its manufacturer, is not guaranteed or endorsed by the publisher.

